# Alexithymia and post-traumatic stress disorder symptoms in Chinese undergraduate students during the COVID-19 national lockdown: The mediating role of sleep problems and the moderating role of self-esteem

**DOI:** 10.3389/fpsyg.2022.1040935

**Published:** 2022-11-11

**Authors:** Yi Zhang, Yijin Zhao, Ting Ni, Jing Chen, Wanjie Tang

**Affiliations:** ^1^School of Economics and Business Administration, Yibin University, Yibin, China; ^2^School of Business, Sichuan University, Chengdu, Sichuan Province, China; ^3^Mental Health Center, Sichuan University, Chengdu, China; ^4^Division of Accounting, Sichuan University, Chengdu, China; ^5^College of Environment and Civil Engineering, Chengdu University of Technology, Chengdu, China; ^6^Department of Psychology and Education, Chengdu Normal University, Chengdu, China; ^7^Institute of Psychiatry, Psychology and Neuroscience, King’s College London, London, United Kingdom

**Keywords:** COVID-19, post-traumatic stress disorder, alexithymia, sleep disturbance, self-esteem

## Abstract

**Objective:**

This study examined whether sleep disturbance was a mediator between alexithymic traits and post-traumatic stress disorder (PTSD) COVID-19 pandemic-related stress symptoms, and explored whether self-esteem moderated the alexithymic contribution to poor sleep and PTSD symptoms.

**Method:**

A representative sample of young adults (*N* = 2,485) from six universities in Southwest China completed online self-report surveys on alexithymia, sleep, PTSD, self-esteem, sociodemographic information, and health-related behaviors.

**Results:**

High alexithymic young adults were found to be more likely to have higher sleep problems and higher PTSD symptoms. The moderated mediation model showed that sleep problems mediated the associations between alexithymia and PTSD symptoms. Alexithymic people with lower self-esteem were more likely to have elevated PTSD symptoms and sleep problems than those with higher self-esteem.

**Conclusion:**

Targeted psychological interventions for young people who have difficulty expressing and identifying emotions are recommended as these could assist in reducing their post-traumatic psychophysical and psychological problems. Improving self-esteem could also offer some protection for trauma-exposed individuals.

## Introduction

Posttraumatic stress disorder (PTSD) symptoms are common psychological problems in people exposed to life-threatening traumatic events (Kessler et al., 2017; Tang et al., 2018; Ding et al., 2022). The COVID-19 pandemic, which was first identified in late 2019, had caused more than 6.48 million global deaths by 29th August 2022. This deadly global pandemic has also resulted in significant public worry and psychological stress (Ji et al., 2021; Liao and Wang, 2021; Tang et al., 2022), such as PTSD symptoms (Johnson et al., 2020; Cénat et al., 2021; Jian et al., 2022). To determine the psychopathological PTSD mechanisms, many studies have sought to identify the psychosomatic symptoms and diathesis factors that contribute to PTSD symptoms, two of which are alexithymia (Badura, 2003; Frewen et al., 2008a; Osimo et al., 2021) and sleep problems (Leskin et al., 2002; Germain, 2013). However, these factors have not been explored in a COVID-19 pandemic context.

Alexithymia, which was originally described as a personality trait and often manifests as an absence of a verbal ability to express emotions (Sifneos, 1973), has many psychological consequences (Scimeca et al., 2014). People with high alexithymia often have externally orientated thinking and an inability to express or recognize their own or others’ emotions. Whether alexithymia is a trait or a state is still being debated; however, many longitudinal studies have found that alexithymia remains relatively stable over time (Salminen et al., 2006; Tolmunen et al., 2011). For instance, in an 11-year follow-up study, alexithymia was found to be a stable personality trait (Tolmunen et al., 2011). As alexithymia is often conceptualized as a lack of emotional self-regulation and competency and an inability to adaptively regulate emotions when under stress, it could be a risk factor for several other mental health problems, such as post-traumatic stress disorder (PTSD). A recent meta-analysis found that people with PTSD tended to have a greater number of alexithymic traits than the general population (Edwards, 2019), and a recent study found that highly alexithymic people were more vulnerable to mental health problems during the COVID-19 pandemic (Osimo et al., 2021).

Litz’s influential PTSD network model (Litz, 1992; Litz et al., 2002) claims that people with PTSD may have some predominant emotional processing schemas that block the expression of emotions, which indicated that the presence of alexithymia may indicate a vulnerability to the development of PTSD (Zlotnick et al., 2001). Frewen et al. (2008b) argued that alexithymic symptoms could predict brain activation in trauma-associated memory areas. Therefore, it is possible that people with alexithymia could be more vulnerable to the development of traumatic stress-related PTSD symptoms.

As there has been little research into the associations between alexithymia and PTSD symptoms in the COVID-19 pandemic context, this study examined the relationship between alexithymia and PTSD symptoms in young adults. While a positive relationship between alexithymia and PTSD symptoms has been assumed, the association mechanism is unclear; therefore, this research also explored the potential relationship mechanism between alexithymia and pandemic stress-related PTSD symptoms.

### Sleep problems as a mediator

Research has shown that sleep problems, such as poor sleep quality and nightmares, tend to occur after trauma exposure and can predict PTSD onset and progression (Ross et al., 1989; Germain, 2013). Previous adolescent and adult studies (Belleville et al., 2009; Brown et al., 2011) have consistently found that sleep problems are strongly correlated with PTSD symptom severity. Prospective and treatment studies have also provided robust evidence for the relationship between sleep problems and PTSD symptoms (Pigeon et al., 2013; van Liempt et al., 2013; Colvonen et al., 2018). Experimental studies that examined the relationship between sleep and PTSD also support the hypothesis that sleep plays an important role in PTSD-relevant processes (Vanderheyden et al., 2015). Therefore, previous studies have strongly indicated that sleep problems may contribute to maladaptive stress and trauma responses and could be a pivotal risk factor for PTSD pathogenesis.

Connections between alexithymia and sleep problems have been found in previous research. For example, independent of mental health problems, alexithymic traits were found to be associated with reduced sleep quality in an adult sample (Murphy et al., 2018), and an alexithymic group scored significantly higher than a non-alexithymic group in a representative undergraduate student sample for a variety of sleep problems, such as insomnia, excessive sleepiness, sleepwalking, and nightmares (Bauermann et al., 2008). When depression and other psychiatric symptoms were controlled for in an outpatient sample, sleep disorders were found to be independently associated with alexithymia (Honkalampi et al., 1999). Several recent studies in adult samples have also found that alexithymic traits were associated with poor sleep quality (Yaşar and Gündoğmuş, 2021; Pojatić et al., 2022).

Although past studies have suggested that sleep problems could be a mediating variable between alexithymia and trauma-related PTSD, few studies had explored the link between sleep problems and pandemic-related PTSD or the underlying psychosocial mechanism of this relationship. For instance, only one study at follow-up found that baseline sleep quality was highly correlated with COVID-19-related PTSD symptoms (Straus et al., 2022). Therefore, to the best of our knowledge, there have been no studies that have explored the underlying psychosocial mechanism between sleep problems and COVID-19-related PTSD symptoms.

Therefore, it is possible that people with high alexithymic traits could be more prone to sleep problems from pandemic stress, which in turn could make them more prone to PTSD symptoms. In other words, sleep disturbances could be a mediator between alexithymia and pandemic-related PTSD symptoms.

### Self-esteem as a moderator

Although alexithymia may be associated with poor sleep quality and PTSD symptoms, different personality traits may result in different reactions. Therefore, it is important to consider the personality moderators that could buffer the relationship between alexithymia and the possible negative outcomes of COVID-19 pandemic stress. As self-esteem, which is a personal construct related to the overall affective evaluation of one’s worth, value, or importance (Blascovich et al., 1991), has been linked to several positive behaviors and psychological manifestations (Rehman et al., 2021; Yan et al., 2021; Iqbal and Dar, 2022; Lee et al., 2022), it could act as a buffer between these adverse effects.

Recent studies have found that self-esteem buffered the mental health symptoms triggered by COVID-19 (Rossi et al., 2020; Lin and Chen, 2021). For example, in a sample of college students, self-esteem was found to increase social support and reduce COVID-19 pandemic stress and related psychological problems (Chen et al., 2021). The anxiety buffering hypothesis theory (Greenberg et al., 1992) primarily sees self-esteem as insulating the self from deep-rooted fears and that this feeling of personal value reduces the susceptibility to mental health problems. Many studies have also found an inverse relationship between self-esteem and psychological distress (Kucharska, 2017; Justo et al., 2018; Zhou et al., 2018), which indicated that self-esteem was possibly protecting against the detrimental psychological effects of the life-threatening pandemic and its associated stressors.

**Figure 1:**
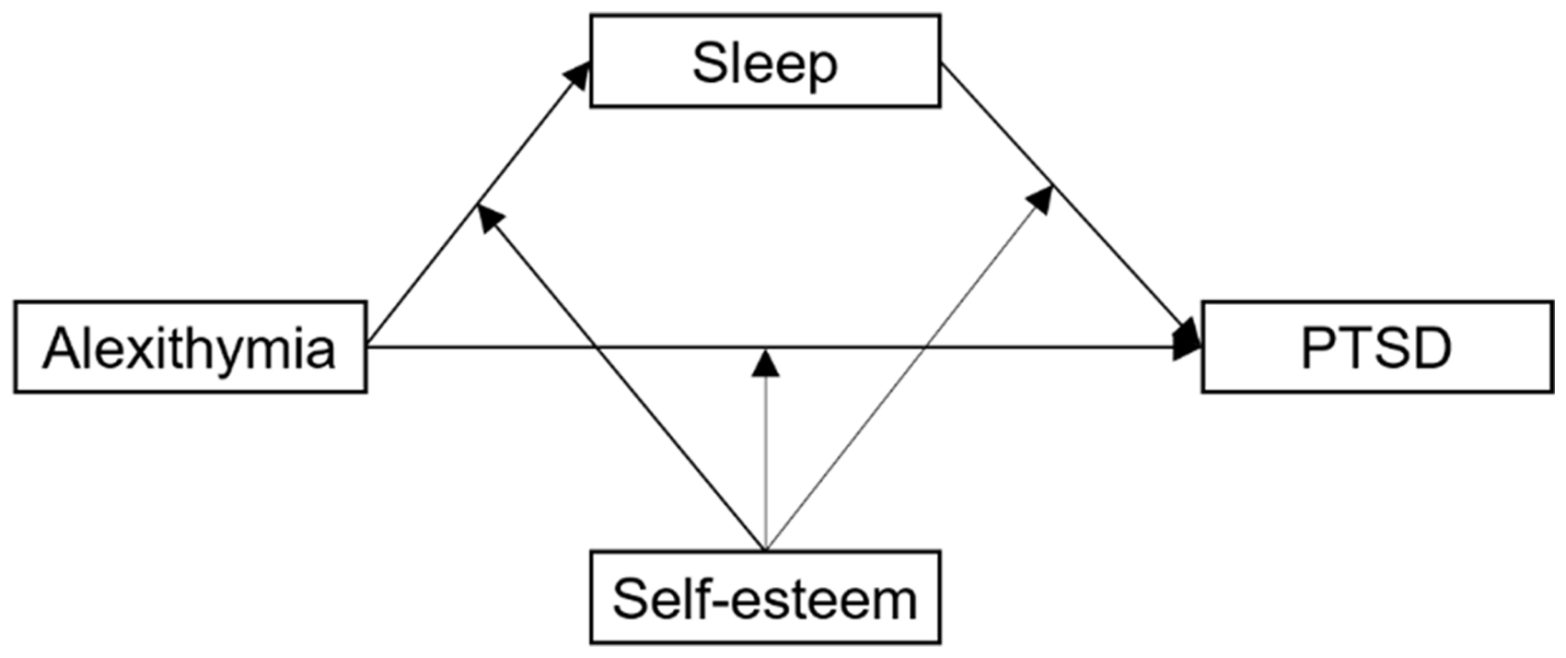
Hypothetical moderated mediation model. PTSD, posttraumatic stress disorder.

Therefore, this study sought to determine the potential mediating roles of sleep disturbance and self-esteem on the relationship between alexithymia and PTSD symptoms during the COVID-19 pandemic, for which the following hypotheses were tested and a mediation model was constructed (see Figure 1).

*Hypothesis 1*: Alexithymia is positively associated with pandemic-related PTSD symptoms.

*Hypothesis 2*: Sleep disturbance mediates the relationship between alexithymia and pandemic-related PTSD symptoms.

*Hypothesis 3*: Self-esteem moderates the direct and indirect effects of alexithymia and pandemic-related PTSD symptoms *via* sleep disturbance.

## Materials and methods

### Study design and procedure

To provide better targeted psychological services for students and increase the understanding of the mental health conditions of university students during the COVID-19 pandemic lockdown, the mental health service departments of six universities in Southwest China agreed to participate in this survey. Undergraduate students from these universities were then invited to participate in an online psychological health survey between 20 February and 27 February 2020, which was during the first COVID-19 wave in China. China went into nationwide lockdown from 23 January to 13 April 2020. Convenience sampling was used in this study, which was approved by the Research Ethics Committee of the Sichuan Psychological Association (2020_12). A random monetary reward (RMB 1–10) was given to motivate student participation in this survey. This study was a secondary analysis, with the recruitment and data collection process detailed in prior work (Tang et al., 2020).

While the students participated anonymously in the survey, they had the option to provide their student numbers so that data could be matched in any following surveys. Students were told that all data would be kept confidential, they should complete the survey based on their first response, and that they could withdraw from the survey at any time if they felt uncomfortable. Because there were 100,000 undergraduates across the six universities, a 95% confidence interval and a 2% error rate margin were taken, which meant 2,345 or more measurements/surveys were needed to have confidence levels of 95% and ensure the real value was within ±2% of the measured/surveyed value. Therefore, using convenience sampling, 120 classes (20 from each university) were selected to participate in the survey. The number of students in each class ranged from 20 to 60; therefore, 3,610 people were invited to participate. The online survey was conducted using the Questionnaire Star Website. First, the survey purpose and informed consent were sent to the students through WeChat groups, after which the students who had signed the online informed consent were forwarded the link and survey QR code through the WeChat group by their coordinator. Participants were required to answer all questionnaire items before moving on to the next item to ensure there were no missing values.

### Instruments and measures

#### Alexithymia

The Chinese version of the Toronto Alexithymia Scales (TAS-20; Taylor et al., 2003; Zhu et al., 2007) was used to measure the alexithymic symptoms, which comprises 20 items across three dimensions; (1) difficulty in identifying feelings, (2) difficulty in describing feelings, and (3) externally oriented thinking; with each item rated on a 5-point scale from 1 = strongly disagree to 5 = strongly agree, with higher scores indicating higher degrees of alexithymia, with the TAS-20 scale cut-off being 61 (Franz et al., 2008). This questionnaire has been proven to have excellent reliability and validity in Chinese populations (Jinyao et al., 2003; Zhu et al., 2007). The McDonald’s *ω* in the current study was 0.86.

### Post-traumatic stress disorder

The PTSD symptom severity in the previous 4 weeks was measured using the Chinese version of the PTSD Check List-Civilian Version (PCL-C), which has had good reliability and validity in Chinese populations (Weathers et al., 1999; Xiaoyun et al., 2007), with a total score of 38 or higher being considered probable PTSD (Dobie et al., 2002). This scale comprises 17 items rated on a 5-point Likert scale from 1 = not at all to 5 = extremely, with higher scores indicating higher PTSD symptoms. The McDonald’s *ω* was 0.92 in the current study.

#### Self-esteem

Self-esteem was measured using the Rosenberg Self-Esteem Scale (Rosenberg, 1965; Du et al., 2016), which comprises 10 items rated on a 5-point Likert scale from 1 (not very true) to 5 (very true), with higher scores indicating higher self-esteem. This scale has been validated in Chinese populations (Du et al., 2012). The McDonald’s *ω* for the scale in this study was 0.78.

#### Sleep disturbance

Sleep disturbance in the previous 4 weeks was measured using the Chinese version of the Pittsburgh Sleep Quality Index (PSQI), which comprises (Buysse et al., 1989) 18 items across seven dimensions: subjective sleep quality; sleep latency; sleep duration; habitual sleep efficiency; sleep problems; sleep medication use; and daytime dysfunction. Each dimension is scored on a 4-point Likert ranging from 0 to 3, with the total score being the sum of the scores from the seven dimensions and the cutoff score being eight (Liu et al., 1996; Kaufmann et al., 2019). This scale has shown satisfactory reliability and validity in Chinese populations (Tsai et al., 2005). The McDonald’s *ω* in the present study was 0.83.

#### Sociodemographic variables and pandemic exposure variables

Demographics such as gender, age, and whether participants were only children were also collected. Pandemic-related exposure variables were measured based on three main questions: someone in the community had been infected; family, friends, and neighbors had been infected; and family, friends, and neighbors had died from COVID-19.

### Data analysis

All statistical analyses were performed using SPSS 22.0. Pearson’s correlation analysis was employed to analyze the correlations between all variables; sleep disturbance, alexithymia, self-esteem, and PTSD symptoms; and mediation analyses were conducted using multiple regression and Hayes’s PROCESS macro for SPSS (Hayes, 2017) in Model 4, with the moderated mediation model being constructed using Hayes’s (2017) PROCESS macro (Model 59).

## Results

### Sample characteristics

While 3,610 undergraduate students were initially invited to participate, only 2,501 completed the surveys, a response rate of 69.3%. Of these, 16 were excluded because of obvious illogical answers, such as all survey choices being the same; therefore, the final sample comprised 2,485 participants, 1,525 of which were female (61.4%), and with the mean age being 19.8 years (SD, 1.55 years; range, 16–27 years; see Table 1).

**Table 1:** Demographic and exposure variables (*N* = 2,485).

Variables	*n*	%
Total	2,485	100
Gender		
Male	960	38.6
Female	1,525	61.4
Age (year)		
16–18	518	20.8
19	648	26.1
20	574	23.1
21	384	15.5
22–27	361	14.5
Only-child status		
Yes	1,061	42.7
No	1,424	57.3
**Type of pandemic exposure**		
Someone in the community infected		
Yes	105	4.2
No	2,380	95.8
Family, friends, and neighbors infected		
Yes	22	0.9
No	2,463	99.1
Family, friends, and neighbors died from COVID-19		
Yes	2	<0.01
No	2,483	>99.99

### Correlations between the key variables

The correlation analyses revealed that PTSD, sleep disturbance, and alexithymia were all significantly and positively correlated (Table 2) and that self-esteem was negatively associated with PTSD, sleep disturbance, and alexithymia. Age, gender, only-child status, and COVID-19-related exposure were not found to have any significant or minor associations with the four main study variables; PTSD, alexithymia, self-esteem, and sleep.

**Table 2:** Correlation coefficients between the main variables (*N* = 2,485).

Variables	1	2	3	4	5	6	7	8	9	10
1. Gender	1									
2. Age	0.46^**^	1								
3. Only-child	−0.05	−0.08^**^	1							
4. Exposure 1^a^	−0.03	−0.02	−0.03	1						
5. Exposure 2^b^	0.01	−0.01	0.03	0.14^**^	1					
6. Exposure 3^c^	−0.01	0.03	0.01	−0.01	0.16^**^	1				
7. PTSD	0.04	0.05	−0.06	0.03	0.03	−0.01	1			
8. Alexithymia	−0.02	−0.03	−0.05	0.04	0.03	−0.01	0.52^**^	1		
9. Self-esteem	0.01	−0.02	0.09^**^	−0.05	−0.02	−0.01	0.33^**^	−0.43^**^	1	
10. Sleep	0.05	0.07^**^	0.01	0.05	0.03	0.01	0.61^**^	0.42^**^	−0.29^**^	1

a “Exposure 1” referred to “Someone in the community infected”.

b “Exposure 2” referred to “Family, friends, and neighbors infected”.

c “Exposure 3” referred to “Family, friends, and neighbors who died from COVID-19.”

*p < 0.05, **p < 0.01. PTSD, post-traumatic stress disorder.

### Mediation effects

In the first step, the multiple regression analyses indicated that alexithymia was significantly associated with PTSD (*b* = 0.52, *p* < 0.001; Table 3). In the second step, the multiple regression analyses indicated that alexithymia was significantly associated with sleep disturbance (*b* = 0.42, *p* < 0.001), and in the third step, when sleep disturbance was controlled for, the multiple regression analyses indicated that alexithymia was still significantly associated with PTSD (*b* = 0.32, *p* < 0.001). Finally, the bias-corrected percentile bootstrap method indicated that the indirect effect of alexithymia on PTSD through sleep disturbance was significant (*b* = 0.20, SE = 0.02 *p* < 0.001; 95%CI, 0.17–0.23).

**Table 3:** Mediation effect of alexithymia on PTSD (*N* = 2,485).

Predictors	Model 1 (PTSD)		Model 2 (Sleep)		Model 3 (PTSD)	
	*β*	*t*	*β*	*t*	*β*	*t*
Alexithymia	0.52	30.18^***^	0.42	23.30^***^	0.32	19.40^***^
Sleep					0.47	28.54^***^
*R* ^2^	0.27		0.18		0.45	
*F*	910.54^***^		543.25^***^		1001.53^***^	

***p < 0.001. PTSD, posttraumatic stress disorder.

### Moderated mediation model

**Figure 2:**
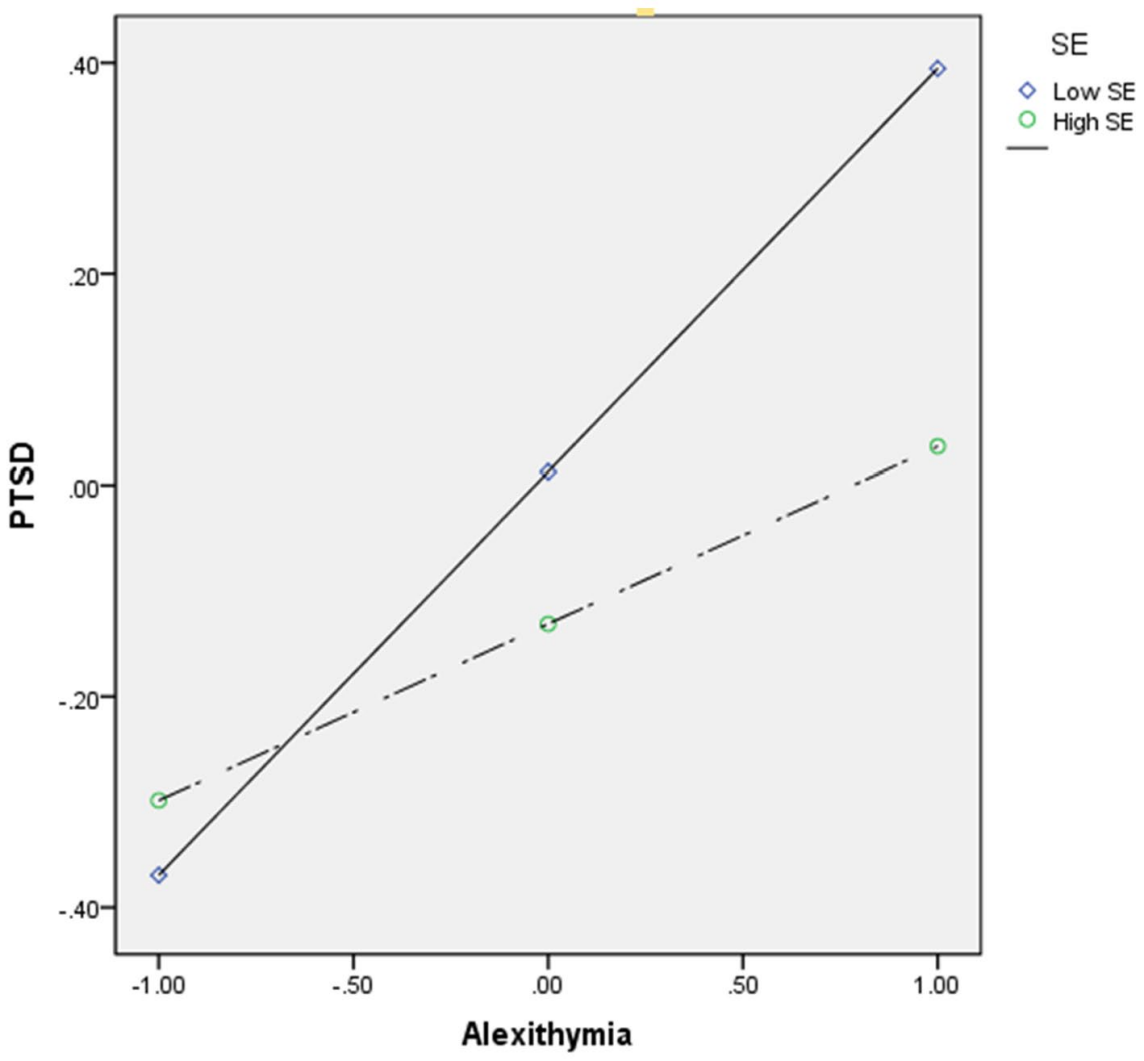
Moderation effect of self-esteem between alexithymia and PTSD. High and low levels of self-esteem represent one standard deviation above and below the mean. SE, self-esteem; PTSD, posttraumatic stress disorder.

As hypothesized, moderated mediation models were established (Table 4). In model 1, the *R*^2^ change was 0.32 and a main effect for alexithymia was found on PTSD, *b* = 0.42, *p* < 0.001, with this effect being moderated by self-esteem, *b* = −0.20, *p* < 0.001(Figure 2).

**Table 4:** Moderated mediation effect of alexithymia on PTSD (*N* = 2,485).

Predictors	Model 1 (PTSD)				Model 2 (Sleep)				Model 3 (PTSD)			
	*β*	SE	*t*	%95CI	*β*	SE	*t*	%95CI	*β*	SE	*t*	%95CI
Alexithymia	0.42	0.019	22.44^***^	0.38–0.46	0.33	0.020	16.65^***^	0.30–0.37	0.27	0.017	15.78^***^	0.24–0.31
SE	−0.13	0.018	−7.28^***^	−0.17 to −0.10	−0.14	0.020	−6.92^***^	−0.18 to −0.10	−0.07	0.016	−4.39^***^	−0.10 to −0.04
Alexithymia * SE	−0.20	0.016	−12.34^***^	−0.23 to −0.16	−0.14	0.017	−8.38^***^	−0.18 to −0.11	−0.11	0.016	−6.64^***^	−0.14 to −0.08
Sleep									0.42	0.017	24.78^***^	0.39–0.45
Sleep * SE									−0.05	0.015	−3.43^***^	−0.08 to −0.02
*R* ^2^	0.32				0.22				0.47			
*F*	393.44^***^				226.95^***^				446.19^***^			

***p < 0.001. PTSD, posttraumatic stress disorder; SE, self-esteem.

**Figure 3:**
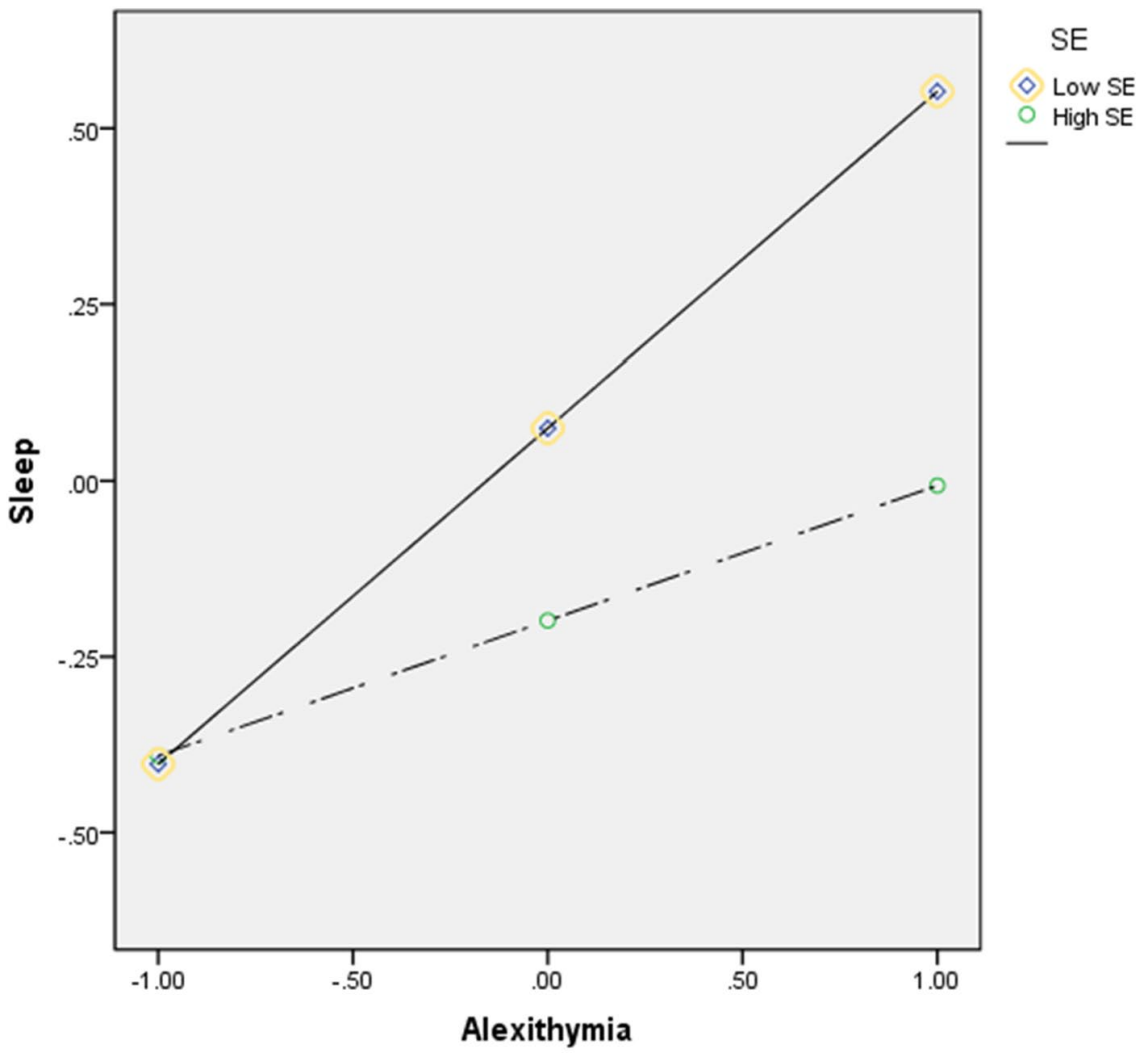
Moderation effect of self-esteem between alexithymia and sleep disturbance. High and low levels of self-esteem represent one standard deviation above and below the mean. SE, self-esteem.

In model 2, the *R*^2^ change was 0.21, alexithymia had a main effect on sleep disturbance, *b* = 0.33, *p* < 0.001, with this effect being found to be moderated by self-esteem, *b* = −0.15, *p* < 0.001(Figure 3).

**Figure 4:**
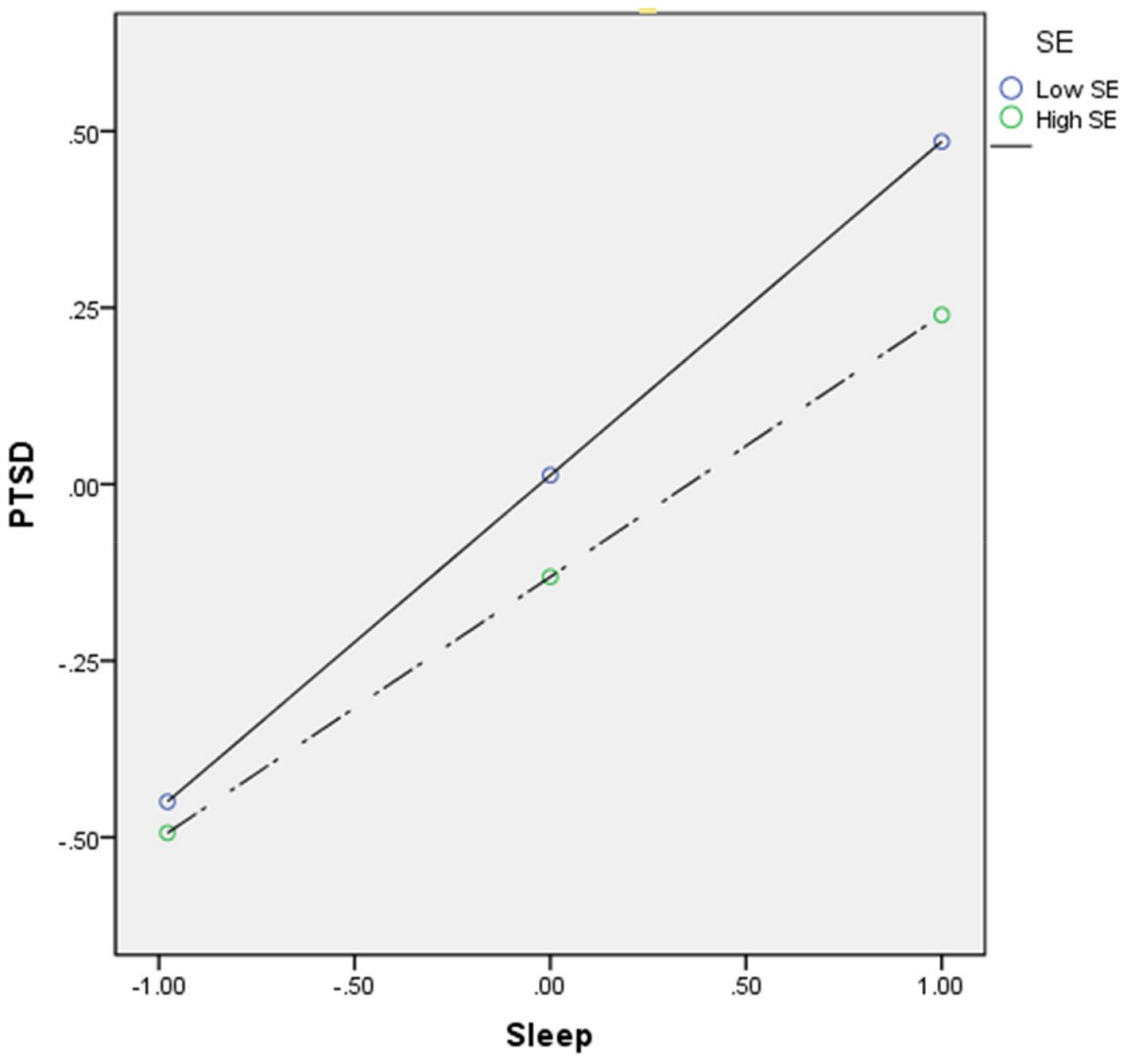
Moderation effect of self-esteem between sleep disturbance and PTSD. High and low levels of self-esteem represent one standard deviation above and below the mean. SE, self-esteem; PTSD, posttraumatic stress disorder.

In model 3, the *R*^2^ change was 0.47, and sleep disturbance had a main effect on PTSD, *b* = 0.42, *p* < 0.001, with this effect being moderated by self-esteem, *b* = −0.05, *p* < 0.001(Figure 4).

## Discussion

To the best of our knowledge, this was the first study to explore the relationships between alexithymia, sleep disturbance, self-esteem, and PTSD symptoms during the COVID-19 pandemic. This study proposed and tested a moderated mediation model to analyze the mechanisms underlying the associations between alexithymic traits and pandemic-related PTSD symptoms in a sample of young adults. The findings supported the hypotheses and suggested that alexithymic traits were highly positively correlated with pandemic-related PTSD symptoms and that sleep disturbance played a mediating role. The direct and indirect associations between alexithymic traits and pandemic-related PTSD symptoms through sleep problems were found to be moderated by self-esteem.

As expected, a significant relationship was found between alexithymia and PTSD symptoms during the COVID-19 pandemic, which was a similar finding to a previous meta-analysis study that found that alexithymic symptomatology was particularly characteristic of individuals with PTSD (Frewen et al., 2008a). The results also aligned with a prospective study that indicated that the alexithymic level was significantly correlated with PTSD symptom severity (McCaslin et al., 2006). The finding that alexithymia was significantly related to PTSD supported the notion that alexithymia is a preexisting personality trait that facilitates trauma-related PTSD symptoms. Litz’s information processing model (Litz, 1992) claims that PTSD symptoms are caused by some defects in the individual information processing schema, which results in emotional obstruction. Therefore, alexithymia, which is difficulties with emotional recognition and a lack of expression, can be explained, that is, young people who experience COVID-19-related stress and who are unable to express their emotions, accumulated fears, and negative emotions may be more likely to develop PTSD symptoms (Velotti et al., 2021). Alexithymic neuroscience studies have also found that as alexithymia symptoms activate the brain areas associated with trauma-related memories, people with alexithymia may be more susceptible to pandemic stress-related PTSD symptoms (Frewen et al., 2008b). This result also supported another neuroscience study that found trait alexithymic symptoms significantly predicted state neural responses to PTSD symptom provocation (Frewen et al., 2006). A recent study also provided evidence that targeted psychotherapy could enhance the ability to identify and express emotions in alexithymic individuals and reduce PTSD symptoms (Zorzella et al., 2020). Therefore, combined with the results in this study, it is possible that early alexithymia identification and early intervention could decrease the effects of the COVID-19 pandemic or other traumatic stresses and the development of more serious PTSD symptoms in young adults.

This study was one of the first to examine the mechanisms underlying the relationships between alexithymia and PTSD symptoms, and therefore, extends existing knowledge by finding that sleep problems partly mediate the association between alexithymic traits and PTSD symptoms. The finding that alexithymic traits are significantly associated with sleep problems supports earlier findings that alexithymia has an independent effect on sleep quality (Kronholm et al., 2008). These findings were also consistent with a recent study that independent of depression and anxiety, heightened alexithymia is associated with poor sleep (Murphy et al., 2018). Parker et al. (1998), concluded that people with high alexithymia and deficits in their reappraisal of negative emotions had poorer maladaptive emotional regulation and immature self-defense styles. An experimental study also found that highly alexithymic individuals with emotion regulation deficits had a greater propensity to elevate their subjective negative affect (i.e., tension and anger) when confronted with stress (Connelly and Denney, 2007), which appeared to raise their vulnerability to sleep problems (Gruber and Cassoff, 2014; Palmer and Alfano, 2017). This study also found that alexithymic traits increased the risk of sleep problems, which in turn elevated the likelihood of developing pandemic-related PTSD symptoms, which was in line with previous studies that found that sleep problems were significantly associated with PTSD symptoms (van Liempt et al., 2013; Fan et al., 2017; Hu et al., 2021). This could explain why people with high alexithymia are more likely to have pandemic stress-related sleep problems and more likely to have PTSD symptoms.

Another significant contribution of this study was that the moderating role of self-esteem was tested in the mediation model, from which it was found that alexithymic people with high self-esteem had fewer sleep problems and lower PTSD symptoms than alexithymic people with low self-esteem. This finding suggested that high self-esteem could to some extent, offset pandemic stress-related sleep problems and PTSD symptom frequencies in people with high alexithymia. High self-esteem was also found to mitigate the negative sleep disturbance effect on PTSD symptoms, which was partly in line with previous studies (Arima et al., 2020; Rossi et al., 2020) that found that self-esteem was a protective factor against adverse psychological consequences in the general population response to the COVID-19 pandemic. The results in this study also supported the stress-buffering effects of the self-esteem model (Rector and Roger, 1997), and offered additional evidence that self-esteem could assist people with strong internal resources to mitigate the negative effects of pandemic stress even when high-alexithymia inflates sleep problems and PTSD symptoms. Therefore, this study’s results highlighted the important role of self-esteem in protecting trauma-exposed individuals from negative consequences and mitigating the effects of personality predisposition on negative mood disorders.

Overall, this study has important implications for preventative mental health interventions in general responses to COVID-19 pandemic stress. As heightened alexithymia may increase the risk of disrupted sleep and PTSD symptoms when suffering from trauma-related stress, the greater a person’s capability to identify, understand, and verbalize emotions and affection, the less likely they are to be overwhelmed by posttraumatic psychophysiological symptoms. Therefore, to reduce the development of these post-traumatic psychophysiological and psychological problems, education and intervention programs are needed that improve people’s abilities to identify and express feelings. Most importantly, this study’s data indicated that sleep problems were a mediator between alexithymia and PTSD symptoms, and elucidated the possible mechanism underlying the effect of alexithymia on mental health problems, that is, improving sleep problems could partially mitigate the possibility of developing pandemic stress-related PTSD symptoms. Finally, because self-esteem was found to act as an alexithymic impact buffer against sleep problems or PTSD symptoms, targeted post-trauma psychological interventions should be focused on promoting self-esteem in the general population.

Regardless of these important findings, this study had several limitations. First, although a mediation moderation model was used in this cross-sectional study, the causality between the psychological alexithymic constructs and PTSD symptoms could not be concluded. Therefore, future longitudinal studies could explore these causal relationships. Second, the use of self-assessment and online surveys as the single psychological measurement method could be seen to be inadequate; therefore, objective measurements and face-to-face interviews could further advance the understanding of these psychological variables and improve reliability and validity. Finally, although the representative sample was recruited from six universities, the unity of the college student population could have affected the applicability and generalization of these results to other groups.

## Data availability statement

The raw data supporting the conclusions of this article will be made available by the authors, without undue reservation.

## Ethics statement

The studies involving human participants were reviewed and approved by the Research Ethics Committee of Sichuan Psychological Association (2020_12). The patients/participants provided their written informed consent to participate in this study.

## Author contributions

YZ, YijZ, JC, and WT wrote the main manuscript text. TN prepared the tables. YijZ prepared the figures. All authors contributed to the article and approved the submitted version.

## Funding

This study is supported by grants from the Science and Technology Department of Sichuan Province (grant no. 2021JDR0346).

## Conflict of interest

The authors declare that the research was conducted in the absence of any commercial or financial relationships that could be construed as a potential conflict of interest.

## Publisher’s note

All claims expressed in this article are solely those of the authors and do not necessarily represent those of their affiliated organizations, or those of the publisher, the editors and the reviewers. Any product that may be evaluated in this article, or claim that may be made by its manufacturer, is not guaranteed or endorsed by the publisher.
